# Damage analysis of *Pochazia shantungensis* (Hemiptera: Ricaniidae) in persimmons

**DOI:** 10.1371/journal.pone.0301471

**Published:** 2024-04-16

**Authors:** Sunghoon Baek, Geonu Lee, Chang-Gyu Park

**Affiliations:** 1 Department of Agriculture and Fisheries Convergence, Korea National University of Agriculture and Fisheries, Jeonju-Si, Republic of Korea; 2 Department of Environmentally Friendly Agriculture, Chungnam Agricultural Research and Extension Services, Yesan-Si, Republic of Korea; National Institute of Agricultural Research – INRA, MOROCCO

## Abstract

An invasive species, *Pochazia shantungensis* (Hemiptera: Ricaniidae), causes serious economic damage to fruit trees. In Korea, this pest is mainly managed using chemical insecticides. However, the management timing and insecticides for *P*. *shantungensis* negatively affect honeybee populations. Thus, this study estimated the decision-making level for *P*. *shantungensis* in persimmons to decrease insecticide application and increase management efficiency. We determined which developmental stage (i.e., egg, nymph, and adult) affected the damage-related factors (numbers of new shoots and fruit formations, and harvest amount) of persimmons using both spatial analyses and linear relationships. The distribution of *P*. *shantungensis* eggs was spatially correlated with the one of persimmon fruit number. However, we did not find any linear relationships between the densities of *P*. *shantungensis* eggs and damage-related factors of persimmons. Instead, we found that the density of *P*. *shantungensis* correlated with the death of oviposited branches. From the developed model of branch death possibility based on egg mass density, 5.75 egg masses per newly developed branch were proposed as the decision-making level. The findings would help increase the efficiency of *P*. *shantungensis* management in persimmon orchards and develop decision-making levels for other insects.

## Introduction

An Asian planthopper, *Pochazia shantungensis* Chou & Lu 1977 (Hemiptera: Ricaniidae), is a highly invasive species originating from China [1, 2]. Although *P*. *shantungensis* is a poor flyer, it has a high affinity for human-modified habitats [2]. Moreover, its host range is broad and its reproductive potential is high [3]. In Korea, this pest is classified as an invasive species and it required only five years since its first occurrence to cause economic damage [1, 2]. Thus, this species has the high potential to become a global problem as it has been observed in Europe [4, 5].

All stages of *P*. *shantungensis* can damage plants; its nymphs and adults can directly cause damage to plants by sucking plant sap, and indirectly by secreting honeydew, while its eggs can not actively cause any damage to plants but egg masses, laid in crevices scratched into new branches, can block the flow of plant saps in newly developed branches (less than one year old) [6]. The activities of *P*. *shantungensis* could affect persimmon development. At the time of nymph emergence, all new shoots have completed their development. Therefore, *P*. *shantungensis* nymphs can not affect new shoot development in plants. The adults emerge in ripening season of fruit trees, when they can feed on plant sap in plant stems or leaves. However, this feeding does not impact the harvest yield in fruit trees [7]. Thus, adult *P*. *shantungensis* do not affect fruit production. However, branches used as oviposition sites by female adults can lose their vitality or die based on the density egg masses by blocking the flow of plant saps [6]. Generally, female adults of *P*. *shantungensis* start to lay eggs in August but oviposition activities peak immediately after the harvesting season in arboreal plants [7]. These oviposited eggs overwinter. Thus, eggs could affect the development of new shoots and fruits, and the harvest amounts in the next year in fruit trees. Owing to these characteristics, damage from *P*. *shantungensis* in Korea has mainly been reported in fruit trees [3].

During the early invasive stages of *P*. *shantungensis* in Korea, this pest caused serious damage. Almost all trees in a few apple orchards and chestnut fields died in Gongju-Si and Yesan-Si in Chungcheongnam-Do, where the first discovery of this species was reported in Korea [8]. However, this serious damage has decreased because efficient management timing has been suggested [7] and more than 100 pesticides for this pest have been registered in Korea [9]. Although insecticide application for *P*. *shantungensis* made it possible to manage this pest, the use of chemical insecticides should be restricted. This is because all registered pesticides of *P*. *shantungensis* are toxic to honeybees and the efficient management timing is slightly earlier than the blooming season of chestnut (*Castanea crenata*) and acacia (*Acacia dealbata*) which are ones among the most important trees for honeybees and the most common trees in forests in Korea [10]. Honeybees are required for fruit trees to produce high-quality fruits.

To solve this dilemma, site-specific management of *P*. *shantungensis* was proposed in chestnut fields [10]. However, the decision-making density for *P*. *shantungensis* has not yet been determined. The application of site-specific management is impossible without decision-making level for this pest. Moreover, decision-making density should be provided to dramatically decrease the use of chemical pesticides and increase the management efficiency of *P*. *shantungensis*.

Reliable decision-making level could be determined by quantifying the yield loss according to the density of a target pest [11]. By increasing its reliability, the decision-making density is generally selected based on multiple criteria including scientific quantification of the crop damage a pest can cause, incorporation of the degree of crop tolerance and resistance to pest damage, practicality of assessing pest density, and the reliability of suggested decision-making level by farmers and agronomists [11–14]. More rational quantification of the crop damage would be possible to consider all available combinations between developmental stages (i.e., egg, nymph, and adult) of *P*. *shantungensis* and damage-related factors (i.e., new shoot numbers, persimmon numbers, and harvested amounts) of persimmons. More samples increase the chances to incorporate the variations of crop tolerance and resistance. Practicality of sampling could be guaranteed by using already developed sampling unit. Finally, reliability of decision-making level could increase by developing it in actual field conditions, not laboratory or controlled conditions. Thus, this study aimed to determine the reliable density in which insecticide application is required for managing *P*. *shantungensis* by meeting the criteria of decision-making level of an insect pest.

## Materials & methods

### Study sites and sampling

Experiments were conducted in an abandoned sweet persimmon (*Diospyros kaki* cv. Dangam) orchard (*N* 35.05878, *E* 128.09183) in Sacheon-Si (SaCS) from 2021 to 2023, a commercial sweet persimmon orchard (*N* 36.09952, *E* 126.78964) in Seocheon-Si (SeoCS) from 2021 to 2023, and a non-commercial sweet persimmon orchard (*N* 35.63537, *E* 127.85165) in Hamyang-Gun (HYG) in 2023. Study sites and sampling methods were determined after having a prior consultation with each farm owner by complying with the experimental guidelines of Korea National University of Agriculture and Fisheries and Rural Development Administration of Korea, and the experimental legislation of Animal and Plant Quarantine Agency of Korea. Each farm owner also allowed to collect persimmon fruits, and informed persimmon cultivars and locations of each cultivar within a field. According to the consultation, the experimental guidelines, and the experimental legislation, we did not take any destructive samples except for harvestable fruits.

In the SaCS persimmon orchard, approximately 600 persimmons grew from downhill to the middle of the hills in a roughly 6,000 m^2^ area. Persimmons (~ 10% of all trees) in the downhill site were managed for personal consumption. Except for these persimmons, this orchard was not managed for more than three years before this study. The upper part of the hill (~ 4,000 m^2^) was irrigated but no planted plants (waste land). Both the orchard and waste land were surrounded by forested areas. Potential hosts (e.g., chestnuts, acorns, and so on) of *P*. *shantungensis* were present in forested areas. The occurrence history of *P*. *shantungensis* was five years [2] and its occurrence amount was fewer than two egg masses per three 60 cm branches (S1 File).

In the SeoCS persimmon orchard, approximately 400 persimmons were managed from the downhill to the middle of the hills in a roughly 4,000 m^2^ area. In the upper part of the hills (~ 4,000 m^2^), another persimmon cultivar (*D*. *kaki* cv. Daebong) and chestnuts were grown without any barrier between them. Both trees are good host plants for *P*. *shantungensis*. These orchards were surrounded by forested areas. The occurrence history was more than 20 years [2] and its occurrence amount was fewer than two egg masses per three 60 cm branches (S1 File).

The persimmon orchard in HYG was newly invaded areas by *P*. *shantungensis*. The orchard was small (~ 400 m^2^) with only 40 persimmon trees. However, an outbreak of *P*. *shantungensis* occurred here with more than 10 egg masses per three 60 cm branch in 2023 (S1 File). Three sides of this orchard were bordered forest except for the front side, where small greenhouse complex was located.

Randomized fixed sampling was applied for the SaCS and SeoCS orchards. The sample sizes were 100 and 114 trees, respectively. First, a persimmon tree was randomly selected. Then, from the selected tree, another tree was randomly selected with approximately 10 m away along the working path within the orchard. The selected trees were marked with a flagging tape (9WKP4, Presco Product Co.; Sherman, TX, USA) and their coordinates were measured using a differentially corrected global positioning system (GPSMAP64, Garmin Ltd.; Olathe, KS, USA). Three branches per tree were randomly selected and marked with the flagging tape at roughly 60 cm away from the branch tip. This sample unit was proposed in a previous study [15]. Thus, total 300 and 342 branches were sampled in both SaCS and SeoCS orchards, respectively. Sampling was conducted in early May, mid-July, and mid-September, corresponding to each developmental stage (egg, nymph, and adult, respectively) of *P*. *shantungensis*. New shoot numbers, persimmon numbers, and harvested amounts per sample unit were also observed at *P*. *shantungensis* egg, nymph, and adult sampling, respectively.

In the HYG orchard, completely random sampling was applied. An ovipositable branch of *P*. *shantungensis* was selected randomly. The number of *P*. *shantungensis* eggs and their survival per branch were observed and recorded. A total of 200 branches were observed on April 4, 2023.

### Spatial relationships between distributions of *P*. *shantungensis* and damage-related factors of persimmons

Spatial associations between the distributions of *P*. *shantungensis* developmental stages (i.e., overwintered egg and nymph) and damage-related factors (number of new shoots, number of fruits, and harvest amounts) of persimmons were analyzed using spatial analysis by distance indices (SADIE). In the abandoned SaCS orchard, the harvest amounts of persimmons were not analyzed because there were no harvestable fruits except in managed areas (~ 10% of all areas). In this orchard, all fruits dropped during ripening before harvesting in unmanaged areas. In the SeoCS orchard, the harvest amounts of persimmons were not analyzed in 2021 because the farm owner randomly harvested fruits before observing the harvested amounts without any marks. The collected persimmon fruits were preserved in a low temperature warehouse in Korea National University of Agriculture and Fisheries.

In SADIE, the index (*X*) of spatial association is the mean of the local correlation coefficients between the clustering indices of the two sets: *X* > 0 for positive spatial association, *X* = 0 for no association, and *X* < 0 for negative association [16]. The associated possibility (*P*) of this index was calculated using randomization tests at *P =* 0.025 (95% confidence level) because the results of spatial association could be positive or negative [10, 16]. The clustering indices of each set for spatial association analysis were also calculated using SADIE. Clustering indices were quantified using the overall aggregation index, *I*_a_ (*D* / *E*_a_), where *D* is the distance to regularity, defined as the minimum total distance that individual samples in an observed arrangement would need to move to result in uniform spatial distribution [17]. *E*_*a*_ is the mean expected distance to regularity [17]. The associated possibility (*P*_*a*_) of this aggregation index was calculated using randomization tests at *P =* 0.05 (95% confidence level) [16]. All SADIE analyses were performed using SADIEShell version 1.22 (Rothamsted Experimental Station; Harpenden, Herts, UK).

Regardless of spatial analyses, all observed datasets were visualized using ArcGIS version 10.1 (ESRI; Redlands, CA, USA). To map each dataset, ordinary kriging was selected and applied to ArcGIS to estimate the values of the unsampled locations.

### Linear relationships between densities of *P*. *shantungensis* egg and damage-related factors of persimmons

Although more than 100 trees were sampled in both SaCS and SeoCS persimmon orchards, the densities of *P*. *shantungensis* nymphs were too low to determine the relationships between the densities of *P*. *shantungensis* developmental stages and damage-related factors in persimmons. Thus, *P*. *shantungensis* eggs were used only to determine linear relationships with damage-related factors. Because three samples were taken in each tree, 300 and 342 samples were taken in the SaCS and SeoCS orchards, respectively. Linear relationships (densities of damage-related factors of persimmons on relative *P*. *shantungensis* egg mass densities) were analyzed using PROC REG [18].

### Death probability of branches according to the densities of *P*. *shantungensis* egg masses

The death of the branches was determined by smoothly bending the tips of the branches. The dead branches were very dry and easily broken even with a small force. The death probability (number of dead branches / observed number of branches, %) of branches on the relative *P*. *shantungensis* egg density was modeled using the Weibull function [19, 20]:

$$f\left(x\right)=100\;\left[1-\text{exp}\;(-{\left[x-a\right]}^{b})\right]$$ (1)

where *f*(*x*) is the death probability of the persimmon branch, *x* is *P*. *shantungensis* egg density per branch, and *a* and *b* are the model parameters. Model parameters were estimated using PROC NLIN [18].

## Results

### Spatial distributions and relationships between distributions of *P*. *shantungensis* and damage-related factors of persimmons

**Fig 1:**
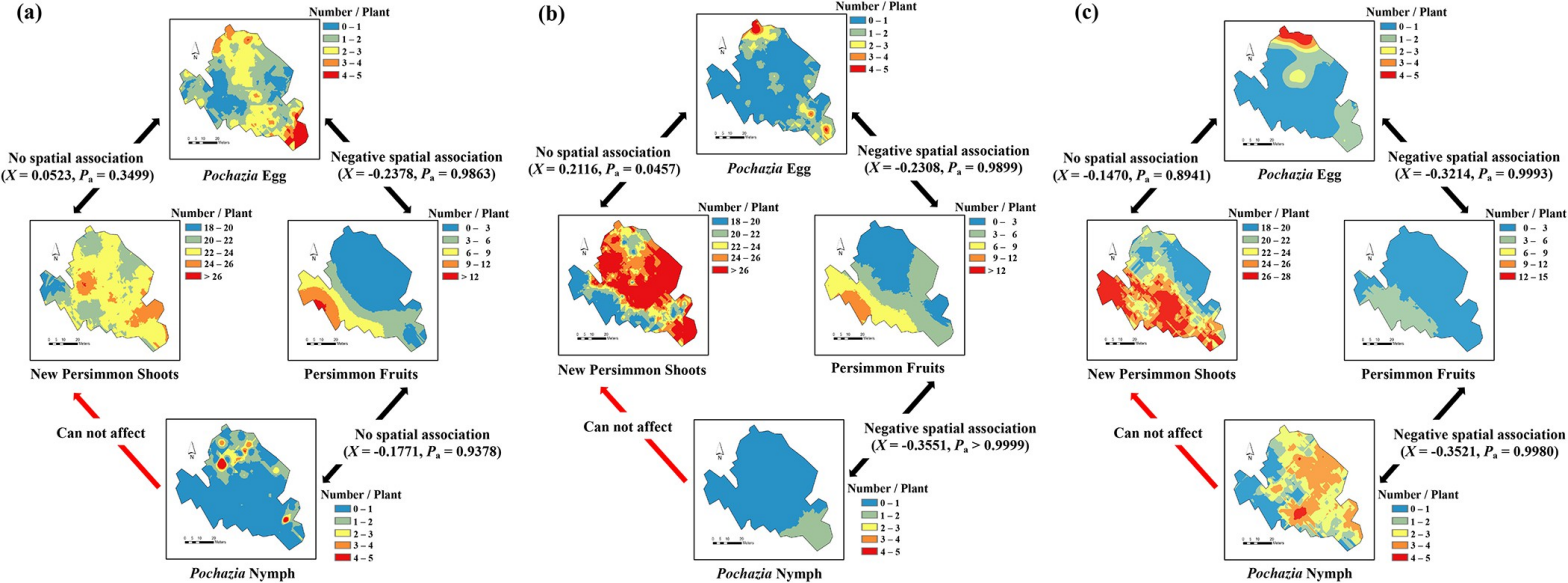
Spatial associations between distributions of *P*. *shantungensis* (i.e., egg masses and nymphs) and damage-related factors (i.e., persimmon new shoot and fruit numbers) in Sacheon-Si in (a) 2021, (b) 2022, and (c) 2023. The sample unit per tree was three 60 cm branch tips.

**Fig 2:**
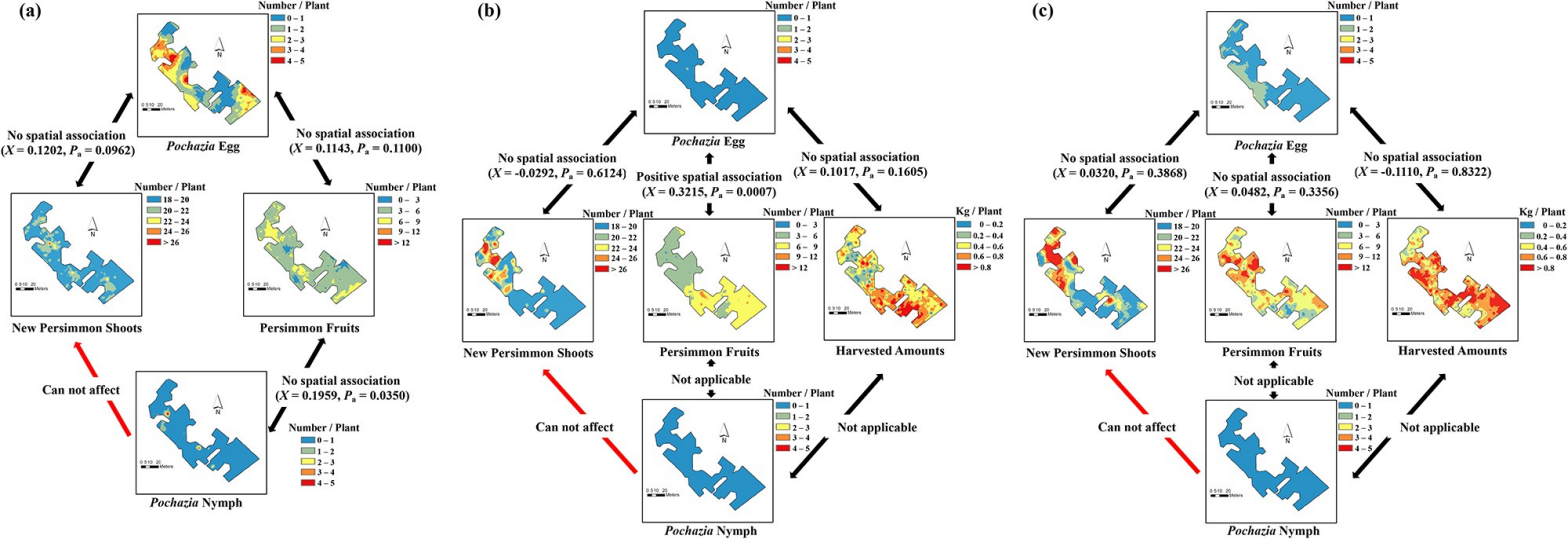
Spatial associations between distributions of *P*. *shantungensis* (i.e., egg masses and nymphs) and damage-related factors (i.e., persimmon new shoot numbers, fruit numbers, and harvest amounts) in Seocheon-Si in (a) 2021, (b) 2022, and (c) 2023. The sample unit per tree was three 60 cm branch tips.

The eggs of *P*. *shantungensis* generally showed an aggregated distribution in the abandoned orchard (SaCS) and a random distribution in the commercial orchard (SeoCS) (Table 1). Its nymphs showed random distributions in both orchards except for SaCS in 2022 (Table 1). Compared with *P*. *shantungensis* egg densities, nymphal densities decreased in both orchards (Table 1). New persimmon shoots developed randomly in 2021 but aggregately in 2022 and 2023 in both orchards (Table 1). However, more persimmon fruits formed in the managed areas than in the unmanaged areas in SaCS (Table 1 and Fig 1). In SeoCS, the distribution of persimmon fruits differed by year (Table 1 and Fig 2). Harvest amounts generally decreased with distance from the orchard owner’s house (the starting point of orchard management) (Table 1 and Fig 2).

**Table 1:** Densities (mean ± SE) and spatial distributions of *P.shantungensis* in Sacheon-Si and Seocheon-Si persimmon orchards at each sampling time.

Target or Year	Stage or Factor	Density	*I*_a_	*P*_a_	Distribution
		(mean ± SE)			
Orchard location: Sacheon-Si					
*P*. *shantungensis*					
2021	Egg	1.8 ± 0.19	1.86	0.0128	Aggregated
2022		0.8 ± 0.15	1.70	0.0128	Aggregated
2023		0.8 ± 0.14	2.07	0.0128	Aggregated
2021	Nymph	0.5 ± 0.17	1.25	0.0897	Random
2022		0.3 ± 0.12	1.82	0.0128	Aggregated
2023		0.5 ± 0.10	1.16	0.1667	Random
Persimmon					
2021	New shoot (ea)	22.6 ± 0.47	0.93	0.5256	Random
2022		24.1 ± 0.77	1.64	0.0128	Aggregated
2023		24.0 ± 0.71	1.46	0.0385	Aggregated
2021	Fruit (ea)	4.4 ± 0.44	2.04	0.0128	Aggregated
2022		4.8 ± 0.42	2.22	0.0128	Aggregated
2023		0.8 ± 0.15	2.01	0.0128	Aggregated
2021	Harvested (g)	-^1^	-	-	-
2022		-	-	-	-
2023		-	-	-	-
Orchard location: Seocheon-Si					
*P*. *shantungensis*					
2021	Egg	1.8 ± 0.20	1.29	0.1410	Random
2022		0.1 ± 0.03	0.66	0.9615	Random
2023		0.7 ± 0.12	1.62	0.0128	Aggregated
2021	Nymph	0.2 ± 0.06	1.01	0.4103	Random
2022		0	-	-	-
2023		0	-	-	-
Persimmon					
2021	New shoot (ea)	18.9 ± 0.31	0.83	0.7179	Random
2022		18.6 ± 0.60	3.10	0.0128	Aggregated
2023		21.4 ± 0.69	2.52	0.0128	Aggregated
2021	Fruit (ea)	4.9 ± 0.25	1.14	0.2308	Random
2022		5.8 ± 0.32	2.76	0.0128	Aggregated
2023		8.6 ± 0.51	1.79	0.0128	Aggregated
2021	Harvested (g)	-	-	-	-
2022		563.3 ± 32.8	1.94	0.0128	Aggregated
2023		682.2 ± 41.28	1.02	0.3590	Random

^1^ No available.

In the SaCS persimmon orchard, there was no spatial relationship between the *P*. *shantungensis* eggs and new persimmon shoots (Fig 1). Even though more *P*. *shantungensis* eggs were found in certain areas within the orchard regardless of the year, new persimmon shoots developed randomly and the highly developed areas changed yearly (Fig 1). However, a statistically significant (*P* < 0.05) negative spatial association between *P*. *shantungensis* eggs and persimmon fruit numbers was observed in three years (Fig 1). In similar to *P*. *shantungensis* eggs, nymph distribution was negatively spatially associated with the distribution of persimmon fruits in 2022 and 2023 (Fig 1).

In the SeoCS persimmon orchard, there were no spatial associations between *P*. *shantungensis* (i.e., eggs and nymphs) densities and damage-related factors (i.e., new shoot number, fruit number, and harvested amount) except for the association between eggs and fruit number in 2022 (Fig 2). In particular, the number of persimmon fruits and harvested amounts were higher in areas where the density of *P*. *shantungensis* was relatively high compared to other areas within the orchard in 2022 (Fig 2).

### Linear relationships between densities of *P*. *shantungensis* egg and damage-related factors of persimmons

**Fig 3:**
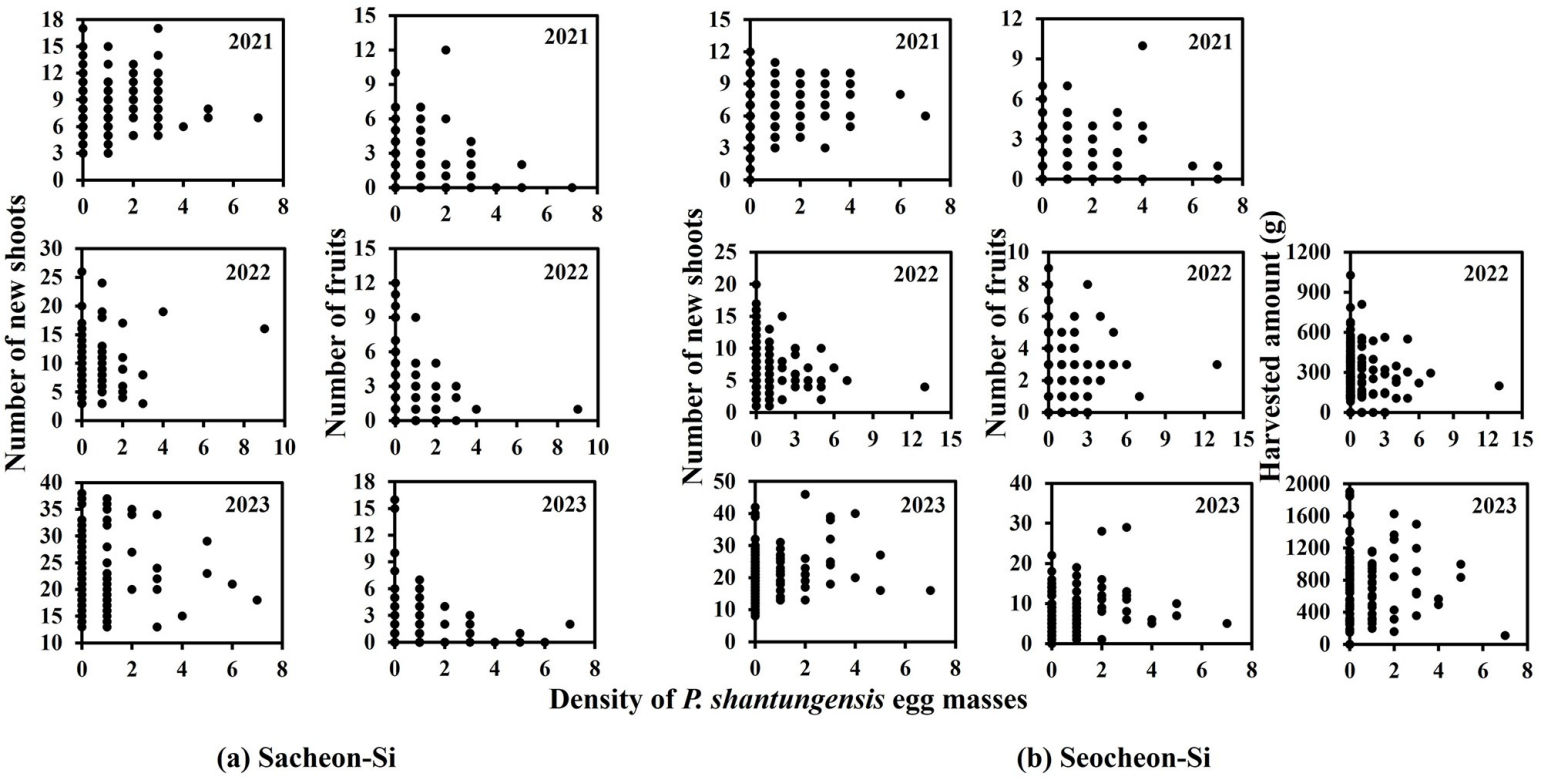
Relationships between densities of *P*. *shantungensis* egg mass and persimmon damage-related factors (i.e., persimmon new shoot numbers, fruit numbers, and harvest amounts) in 2021, 2022, and 2023 in (a) Sacheon-Si and (b) Seocheon-Si. The sample unit per tree was three 60 cm branch tips.

In both orchards, statistically significant (*P* < 0.05) linear relationships between densities of *P*. *shantungensis* egg and damage-related factors of persimmons were only found in two among nine sets (Table 2). For the significant relationships, the developed linear models explained less than 5% of the variances (Table 2). According to the increase in *P*. *shantungensis* egg density, an increase in damage to persimmons was not observed in this field study (Fig 3).

**Table 2:** Statistical summary of the linear relationships between densities of *P.shantungensis* and damage-related factors (i.e., persimmon new shoot numbers, fruit numbers, and harvest amounts) of persimmons in Sacheon-Si and Seocheon-Si.

Site	Year	Damage-related factor	df	*F* value	*P* value	*r*^2^ value
Sacheon	2021	New shoot number	1, 298	6.68	0.0102	0.0219
Sacheon	2021	Fruit number	1, 298	0.04	0.8323	0.0002
Sacheon	2022	New shoot number	1, 298	13.29	0.0003	0.0427
Sacheon	2022	Fruit number	1, 298	0.08	0.7816	0.0003
Sacheon	2023	New shoot number	1, 298	1.17	0.2830	0.0118
Sacheon	2023	Fruit number	1, 298	3.93	0.0502	0.0386
Seocheon	2021	New shoot number	1, 340	3.67	0.0563	0.0107
Seocheon	2021	Fruit number	1, 340	0.84	0.3607	0.0025
Seocheon	2022	New shoot number	1, 340	0.81	0.3683	0.0024
Seocheon	2022	Fruit number	1, 340	1.94	0.1646	0.0057
Seocheon	2022	Harvested amount	1, 340	1.94	0.1644	0.0057
Seocheon	2023	New shoot number	1, 340	3.80	0.0536	0.0328
Seocheon	2023	Fruit number	1, 340	1.76	0.1877	0.0154
Seocheon	2023	Harvested amount	1, 340	0.18	0.6732	0.0016

### Death probability of branches according to the densities of *P*. *shantungensis* egg masses

**Fig 4:**
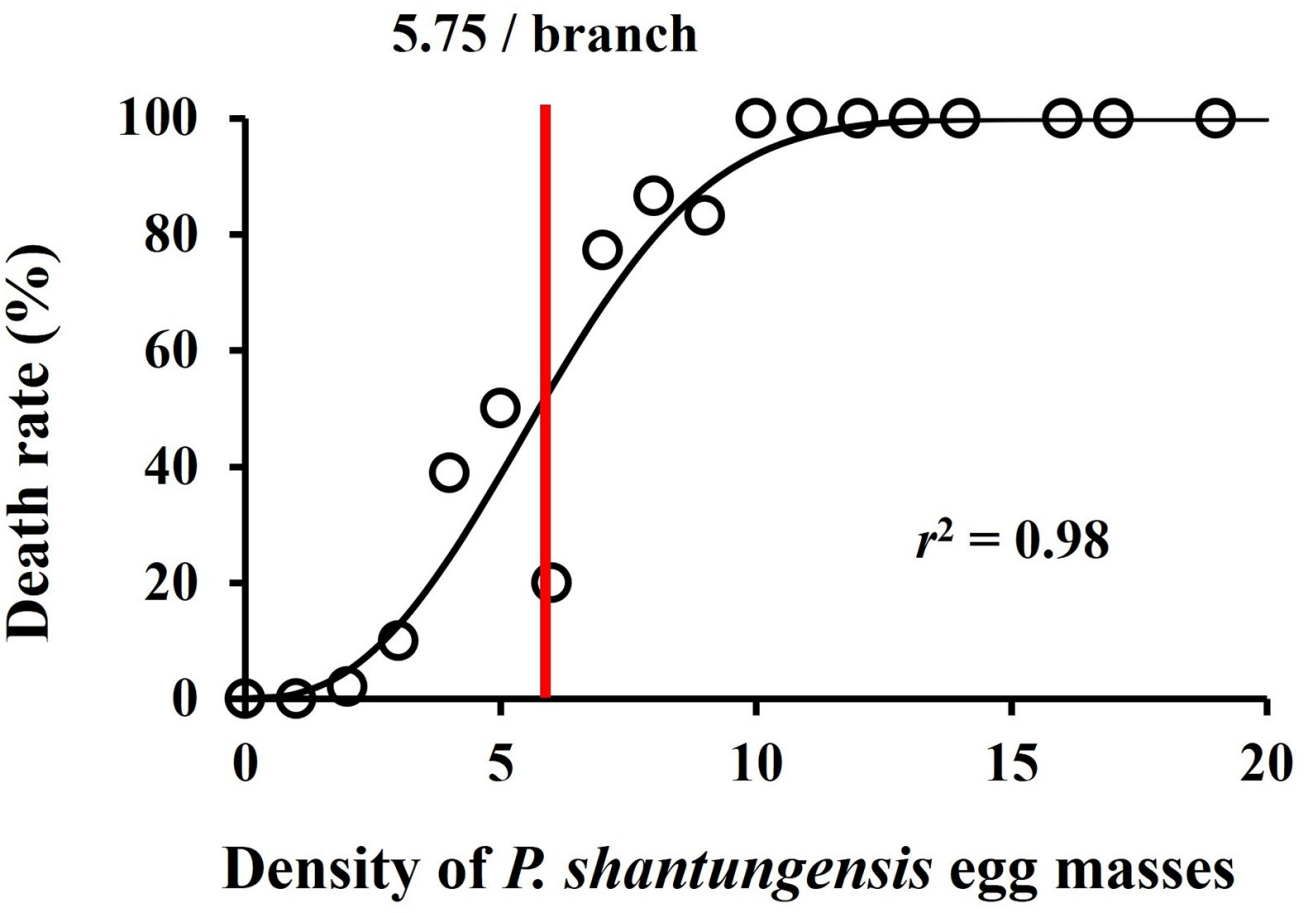
Death probability (%) of newly developed branches of persimmons according to the densities of *P*. *shantungensis* egg masses. The red line in the figure indicates the density of *P*. *shantungensis* egg masses at a 50% branch death possibility.

The Weibull function well predicted death probability (%) of persimmon branches according to the density of *P*. *shantungensis* egg masses (Fig 4, *F* = 453.76; df = 1, 16; *P* < 0.0001). The parameters (estimate ± SEM) in this model were 6.6554 ± 0.3588 and 2.5067 ± 0.4712 for *a* and *b*, respectively. A branch death rate of 50% occurred with 5.75 egg masses per newly developed branch (Fig 4).

## Discussion

The decision-making density of *P*. *shantungensis* could not be determined from changes in the amounts of harvested persimmons according to their densities. In the commercial orchard in this study, harvested persimmon amounts were affected more by farm owners’ activities than by *P*. *shantungensis* densities. Thus, the decision-making density of *P*. *shantungensis* needs to be determined regardless of farm owners’ activities. One of the methods in *P*. *shantungensis* is to find out the density of egg masses required to kill the newly developed branches. The branch death probability model developed in this study could make this prediction based on the density of *P*. *shantungensis* egg masses.

It is known that there are positive spatial relationships among all developmental stages of *P*. *shantungensis* within a field [10]. Moreover, all developmental stages of *P*. *shantungensis* could damage persimmons. These ecological characteristics of *P*. *shantungensis* make us to predict that the spatial relationships between distributions of *P*. *shantungensis* and damage-related factors would be similar regardless of its developmental stage (i.e., egg, nymph, and adult). However, nymphs of *P*. *shantungensis* were rarely found in arboreal fields because the nymphs prefer herbaceous rather than arboreal plants [7]. This phenomenon would explain the lack of a spatial relationship between distributions of nymphs and formed fruits compared to eggs. However, adult female of *P*. *shantungensis* generally lay eggs only on the newly developed branches of arboreal plants [7]. These eggs block the flow of plant sap in the newly developed branches of trees [6]. As egg mass increases, the flow of plant sap in the branches slows, and eventually, the heavily ovipositied branches die. Indeed, the main damage caused by *P*. *shantungensis* is due to oviposition [7]. This could be the main reason why the egg distribution of *P*. *shantungensis* frequently showed statistical (*P* < 0.05) relationships to the distribution of fruit numbers in persimmon orchards in this study.

Interestingly, spatial associations between distributions of *P*. *shantungensis* egg and persimmon fruits showed contrasting results in the two orchards: negative spatial association in the abandoned orchard; no and positive associations in the commercial orchard. Moreover, *P*. *shantungensis* eggs did not spatially affect new shoot development or the harvested amounts of persimmon in both orchards. The decreased flow of plant saps in persimmons might not affect the number of newly emerged shoots but affect the development of leaves from the shoots. In abandoned orchards, this damage would be directly reflected in the number of fruits formed in damaged branches because no fertilizers were applied. However, fertilizers were applied and there were averagely 6.6 branches per sample unit in the commercial orchard. A small decrease in the flow of plant saps would not affect the leaf development. Even if one or two newly developed branches die in the main branch, other branches could produce more fruits than regular branches. It was also known that adult females could distinguish more vigorous trees [10]. Thus, areas with more eggs within an orchard could produce more fruit. Although more fruits were formed, the number of ripened fruits was generally controlled by thinning the fruits. Therefore, increased fruit formations did not affect the amount of harvested fruit in the commercial field.

In the spatial analyses, the distribution of *P*. *shantungensis* egg was spatially associated with the distribution of the formed fruits. However, there was no statistically significant (*P* < 0.05) linear relationship between egg and persimmon fruit densities in either the abandoned or commercial orchards. In this study, the areas with relatively high densities of *P*. *shantungensis* eggs were very small. Moreover, variations in the density of persimmon fruits per sample unit were high. The small number of samples with high densities of *P*. *shantungensis* eggs and the high variation between samples of persimmon fruits would result in an insignificant (*P* > 0.05) relationship between egg density and formed fruit number.

Fortunately, any transmissions of plant disease by *P*. *shantungensis* have not been reported yet. This pest affects vitality or death only in newly developed branches on which fruits are formed in fruit trees [7]. In fruit trees, dead branches can not revive or produce fruit. In practical aspects, the management goal for *P*. *shantungensis* should be to control the number of newly produced branches on a tree. By controlling the density of *P*. *shantungensis* under its density required to kill newly developed branches, economic damages could be prevented and tree vigor could be maintained. This study provided the model for the death probability (%) of persimmon branches according to the densities of *P*. *shantungensis* egg masses. At a branch death probability of approximately 50%, small density increases in *P*. *shantungensis* egg masses caused a sharp increase in branch mortality. Thus, *P*. *shantungensis* needs to be controlled under 5.75 egg masses per newly developed branch. The sample unit of *P*. *shantungensis* was already suggested by the previous study [11] as the 60 cm branch tip. The decision-making level of *P*. *shantungensis* in persimmons was roughly 5.5 egg masses per the sample unit, 60 cm branch tip, when the level was transformed from the whole branch to the sample unit using the relationship, cumulative egg mass proportions (%) of *P*. *shantungensis* according to distances from the tip within a branch in the previous study [15].

The results of this study indicated that the densities of *P*. *shantungensis* egg masses could affect the number of formed fruits in persimmons, but the changes were not related with the decreases of harvested amounts in persimmons. However, the densities of *P*. *shantungensis* egg masses affected the death probability of newly developed branches. To prevent a sharp increase in branch mortality, *P*. *shantungensis* should be controlled under 5.75 egg masses per newly developed branch. This decision-making density of *P*. *shantungensis* would help increase the efficiency of *P*. *shantungensis* management in orchards by preventing prophylactic insecticide application [21–24], decreasing the likelihood of resistance developing [25], and increasing direct and indirect yield [26–30]. Moreover, this study provides a new approach for determining the decision-making density of fruit trees. In the areas of damage analyses, the results estimated under these highly controlled conditions such as laboratories or experimental farms has made a difference from those in commercial fields. The suggested methodology should make possible to estimate reliable decision-making densities in other insect pests under field conditions.

## Supporting information

S1 File Full data of this experiment. (XLSX)
